# Supplementary material for: Priming with Porcine Blood Polypeptide Enhances Salt Tolerance in Wheat Seedlings
Source: Plants (Basel). 2025 Sep 25;14(19):2968. doi: 10.3390/plants14192968 (PMC12526353; doi:10.3390/plants14192968)
Supplement: Supplementary file 1 [file plants-14-02968-s001.zip › Supplementary Figure S1.pdf]

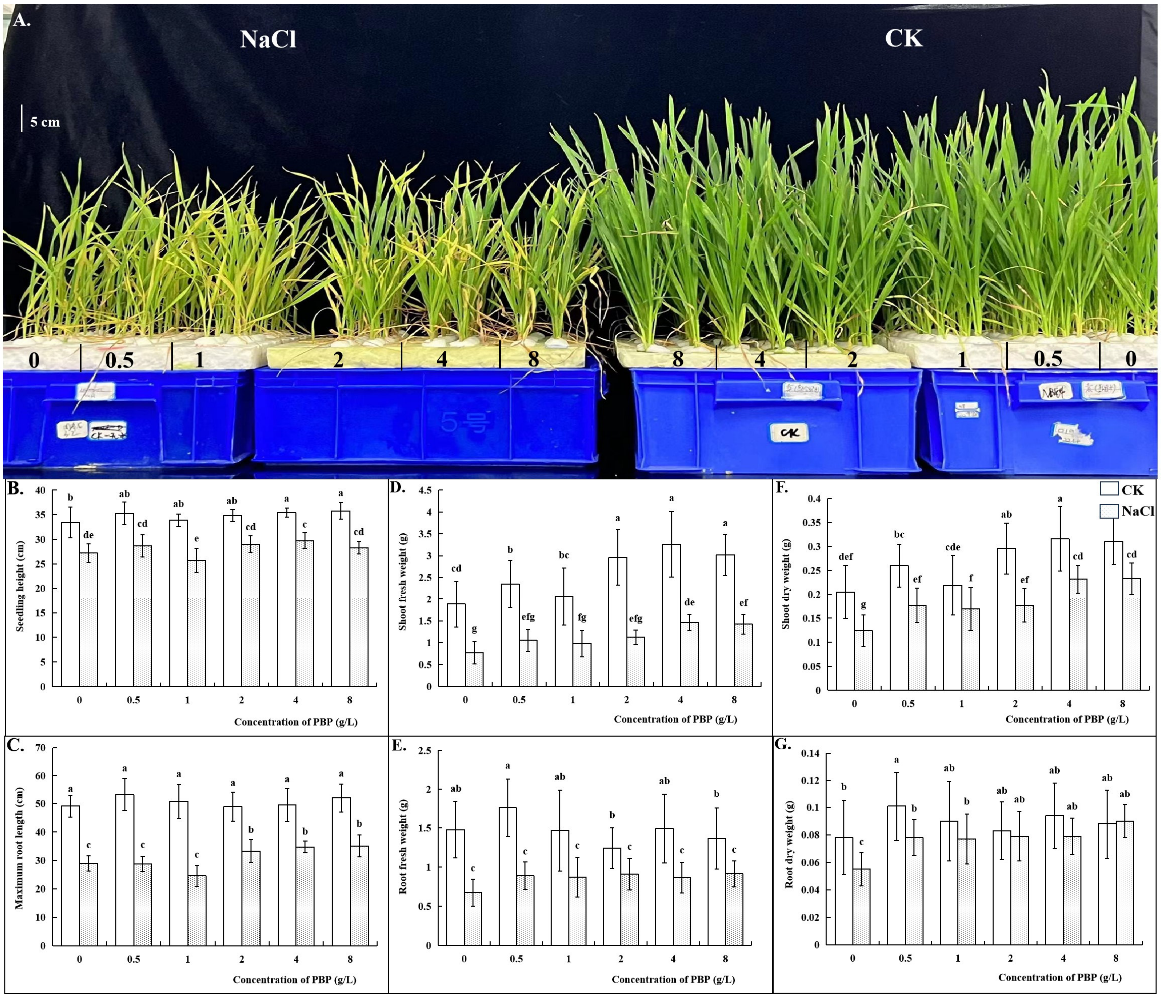

Supplementary Figure S1. Effects of different PBP concentrations on wheat seedlings under salt stress. (**A**) The overall growth of wheat seedlings under normal/salt stress conditions with/without PBP priming, (**B**) seedling height (SH), (**C**) maximum root length (MRL), (**D**) shoot fresh weight (SFW), (**E**) root fresh weight (RFW), (**F**) shoot dry weight (SDW), and (**G**) root dry weight (RDW). Bars are the standard deviations (SDs) of three independent replicates ($n = 3$). Error bars labeled with different letters indicate significant differences at $p < 0.05$ between treatments according to Duncan's test.
